# Depth of Cure of Resin-Based Composites Irradiated With Three Types of Light-Curing Units at Different Output Intensities

**DOI:** 10.7759/cureus.71825

**Published:** 2024-10-19

**Authors:** Toshiyuki Okuse, Keigo Nakamura, Saho Komatsu, Aya Miyashita-Kobayashi, Akiko Haruyama, Akio Yamamoto, Atsushi Kameyama

**Affiliations:** 1 Department of Cariology, Endodontology and Periodontology, School of Dentistry, Matsumoto Dental University, Shiojiri, JPN; 2 Department of Operative Dentistry, Cariology and Pulp Biology, Tokyo Dental College, Tokyo, JPN; 3 Department of Oral Diagnosis and Comprehensive Dentistry, Matsumoto Dental University Hospital, Shiojiri, JPN

**Keywords:** depth of cure, irradiation distance, light-curing unit, output intensity, resin-based composite, shade

## Abstract

Aim

Dental light-curing units (LCUs) are used at Matsumoto Dental University Hospital (MDUH). However, the time of installation of the same type of light irradiator varies, which affects the output intensity of the LCU. The purpose of this study was to evaluate the performance of LCU with different output intensities by comparing the depth of cure (DOC) of resin-based composites (RBCs).

Materials and methods

The output intensities of three types of LCUs, namely Pencure 2000(Morita, Osaka, Japan)*, *DC BlueLEX Plus (Yoshida, Tokyo, Japan)​​​​​​​, and Candelux* *(Morita, Osaka, Japan), were measured using a commercial dental radiometer, namely Bluephase Meter II (Ivoclar Vivadent, Schaan, Liechtenstein). The units with the highest and lowest output intensities were selected and used. The RBC, either Body A3 or Opaque A3 (Premise, Kerr, Brea, USA)​​​​​​​, was inserted into a cylindrical mold with an inner diameter of 4 mm and depth of 8 mm, and light irradiation was performed using an intervening polyester strip for 30 s. After removing the unpolymerized portions of the RBC from the mold with a plastic spatula, the long axis of the cured portion was measured with a digital caliper (*n*=10).

Results

The highest DOC was observed when the RBCs were cured with Pencure 2000 at 1513 mW/cm^2^, the highest output intensity. The DOC was significantly greater when the LCU was positioned at 0 mm than at 8 mm from the RBC surface, and the DOC of Body A3 was greater than that of Opaque A3 (*p*<0.05). Moreover, a positive correlation was observed between output intensity and DOC. The output intensity of LCUs in the same model also varied, which affected the DOC.

Conclusion

Increasing the output intensity at the tip of the light guide of the LCU also increased the DOC of the RBC. Increasing the irradiation distance from 0 mm to 8 mm decreased the DOC of the RBC. The DOC of the opaque-shade RBC was smaller than that of the body-shade RBC when curing was conducted with the same LCU.

## Introduction

Resin-based composites (RBCs) have been widely applied for direct restorations of both anterior and posterior teeth in current dental practice. Current RBCs and adhesive materials have a wide range of color tones, thereby enabling restorations in esthetic harmony with the surrounding tooth structure. RBC restorations are generally executed according to the biomimetic approach in which two RBCs are layered. One RBC is opaque and slightly yellowish, approximating the color of dentin, while the other RBC is white and transparent, approximating the color of enamel. RBC restorations using the biomimetic approach can achieve high clinical results both esthetically and functionally with minimal tooth material removal [1].

RBC restorations are widely accepted by dentists because of their improved handling. In the past, only paste-type RBCs were available, and these were generally applied using specialized instruments. Currently, various flowable RBCs are available, including injectable RBCs that can be injected directly into the cavity from a syringe [2]. By combining RBCs with different flowability for molar cavities with relatively large tooth defects, restorations are simple to execute and have few gaps [3].

In the last century, quartz-tungsten-halogen (QTH) lamps have been used as the light source to initiate RBC polymerization, and the emergence of high-brightness blue light-emitting diodes (LEDs) in 1991 has triggered a series of developments in dental LED light-curing units (LCUs) [4-6]. Early models of dental LED LCUs have very low power outputs [7], while many later models have high power outputs [8,9]. However, because some QTH LCUs are installed as integral components of dental units, they have not been completely replaced by LED LCUs. In fact, LED LCUs are predominantly used in hospitals, although QTH LCUs built into dental units are still in use. LED LCUs in use today are installed at different times, and the newest LCUs are not always in use. The output intensity of LCUs of the same model also varies depending on the time of installation, frequency of use, and degree of maintenance [10,11]. Curing of RBCs with low-power LCUs results in inadequate polymerization, and thus the resulting RBC lacks the physico-chemical properties for optimal clinical outcomes [12]. Moreover, when RBCs are filled into dentin defects of molars, polymerization is difficult because the LCU cannot be placed close to the RBC [13].

The purpose of this study was to compare the depth of cure (DOC) of two types of RBCs with different opacities cured using two types of dental LED LCUs and one type of dental QTH LCUs. Irradiation of the RBCs was performed at two different irradiation positions. Specifically, the LCU was placed close to the surface of the RBCs (irradiation distance of 0 mm) and the LCU was moved 8 mm from the surface of the RBCs (irradiation distance of 8 mm). The null hypotheses of this study are: 1) differences in output intensity and irradiation distance of the LCU tip do not affect the DOC of RBCs, and 2) the shade of the RBC does not affect the DOC of RBCs.

## Materials and methods

Table 1 shows the specifications of the LCUs used in this study, including their serial numbers. As of August 2020, Matsumoto Dental University Hospital housed 38 units of nine models of LCUs, including two LED LCUs, namely Pencure 2000 (Morita, Osaka, Japan) and DC BlueLEX Plus (Yoshida, Tokyo, Japan), and one QTH LCU, namely Candelux (Morita). The output intensity of the LCUs was measured using a dental radiometer (Bluephase Meter II, Ivoclar Vivadent, Schaan, Liechtenstein). Among the LCUs of each model, the one with the highest and the one with the lowest output intensity, measured with the dental radiometer, were selected and used in this study. 

**Table 1:** Output intensities of light-curing units measured using Bluephase Meter II * LED: light-emitting diode. QTH: quartz-tungsten-halogen ** Used in this study

Light-curing unit	Type*	Serial no.	Output intensity (mW/cm^2^)	
(Manufacturer)				
Pencure 2000	LED	SN7156	1320	
(J. Morita Mfg.)		SN7144	1473	
		SN7157	1460	
		SN6024	1100	
		SN7146**	1030	Lowest
		SN7148	1293	
		SN7145	1290	
		SN2736**	1513	Highest
		SN3801	1070	
		SN6399	1473	
		Mean ± S.D.	1302 ± 182	
DC BlueLEX Plus	LED	Undescribed	623	
(Monitex Industrial/Yoshida)		Undescribed	643	
		Undescribed**	320	Lowest
		Undescribed	540	
		Undescribed**	740	Highest
		Mean ± S.D.	573 ± 158	
Candelux (J. Morita Mfg)	QTH			
(Resin mode)		Undescribed	430	
		Undescribed**	323	Lowest
		Undescribed**	460	Highest
		Undescribed	393	
		Mean ± S.D.	402 ± 59	

**Figure 1:**
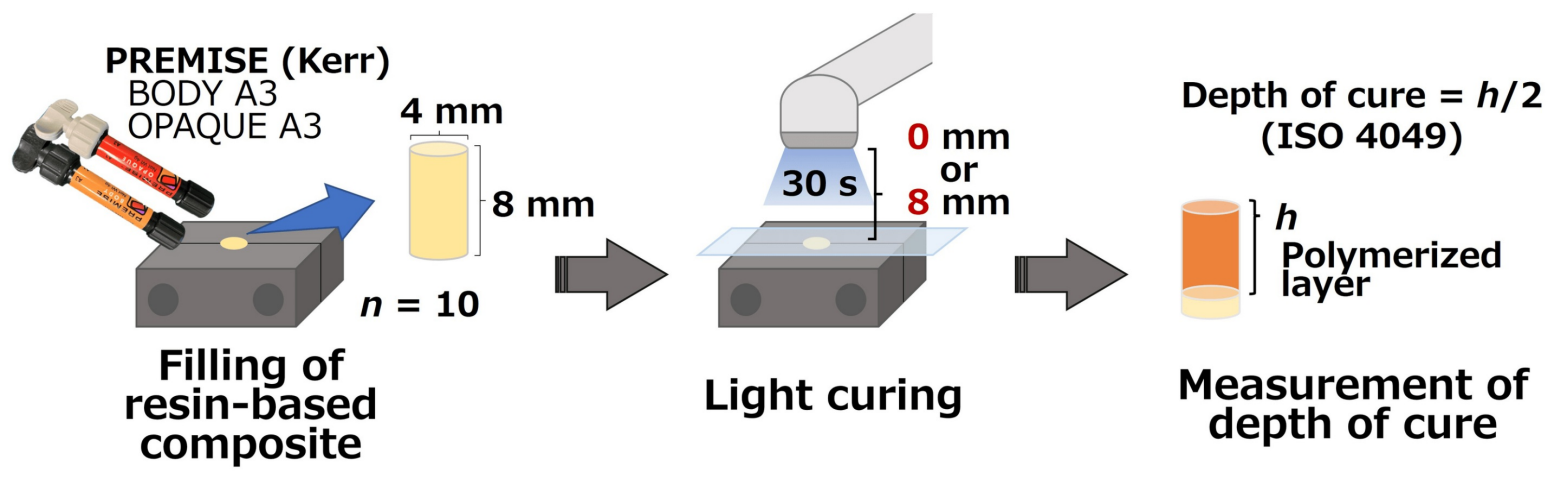
Schematic illustration of the experimental procedure.

The experimental procedure is shown in Figure 1. A cylindrical segmented stainless steel mold (diameter of 4 mm and depth of 8 mm) was sprayed with a mold release agent (Daifree GA-3000, Daikin Industries, Osaka, Japan) and then filled with a slightly larger amount of an RBC, either Body A3 or Opaque A3 (Premise, Kerr, Brea, CA). A glass plate was placed on a polyester strip (thickness of 50 µm) (Epitex, GC, Tokyo, Japan), and pressure was applied by hand. The glass plate was then removed and irradiated with light for 30 s. The output intensity of the LCUs was checked as necessary with Bluephase Meter II (Ivoclar Vivadent) to confirm that there was no significant change from the previously measured value.

The unpolymerized portion of the RBC sample was removed from the mold with a plastic spatula. Then, the long axis of the cured portion was measured with a digital caliper (n=10). Light irradiation was applied to the polyester strip in contact with the mold (0 mm) and to the polyester strip located 8 mm from the mold (8 mm).

Data were subjected to factorial analysis of variance (ANOVA) and compared between groups using the Tukey-Kramer test (p<0.05). Statistical analysis was performed using SPSS Version 18 for Windows (SPSS Inc., Chicago, USA). Correlations between the output intensity and DOC were also examined.

## Results

**Figure 2:**
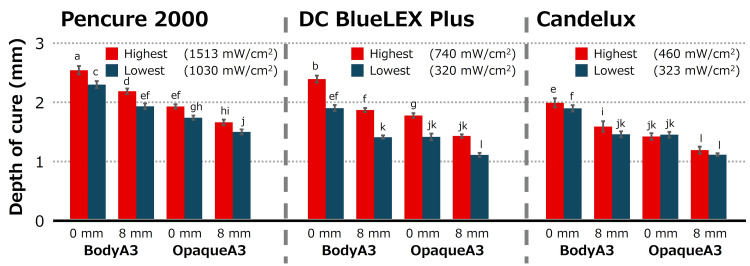
Depth of cure of each experimental group. Same letter indicates the absence of statistically significant difference (p > 0.05, Tukey–Kramer test). Same letter indicates absence of statistically significant differences (*p* > 0.05, Tukey-Kramer test)

The DOC values of resin-based composites cured with each LCU are shown in Figure 2. Four-way ANOVA of the data showed that the following factors (p<0.05) had a statistically significant effect on the DOC: light-curing unit, serial number, composite shade, and irradiation distance. Pencure 2000 provided the highest DOC, followed by DC BlueLEX Plus and Candelux (p<0.05). Moreover, the DOC of Body A3 was higher than that of Opaque A3. The highest and lowest DOC values provided by Pencure 2000 and DC BlueLEX Plus were significantly different. However, when Opaque A3 was irradiated with Candelux at either 0 mm or 8 mm, there was no significant difference between the highest and lowest DOC values.

**Figure 3:**
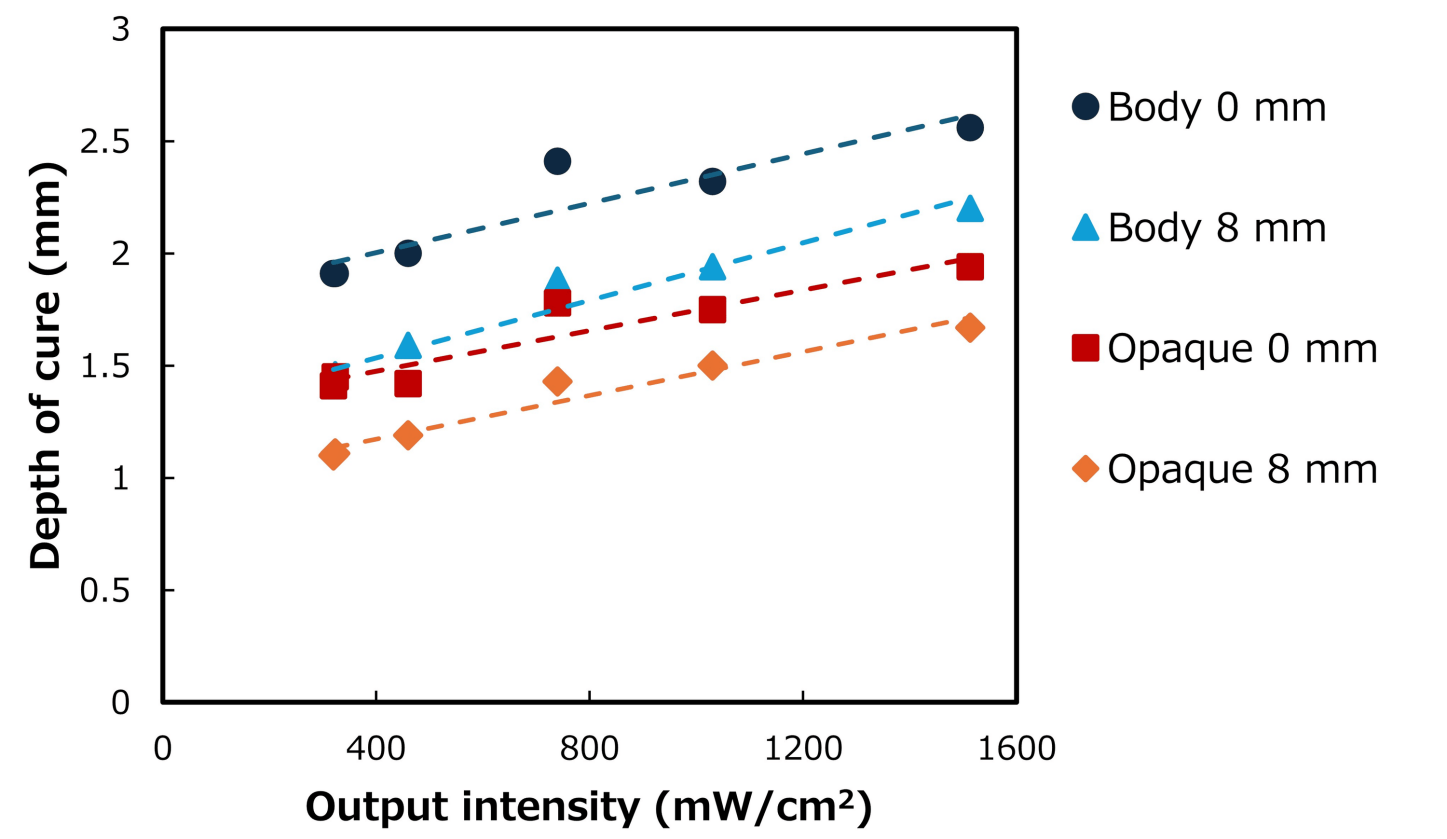
Correlation between the depth of cure and light output intensity.

The correlation between LCU output intensity and DOC is shown in Figure 3. This correlation was represented by y = 0.0006x + 1.7826 (R² = 0.8517) for irradiation of Body A3 at 0 mm and by y = 0.0006x + 1.2782 (R² = 0.9483) for irradiation of Body A3 at 8 mm. Similarly, this correlation was represented by y = 0.0005x + 1.2954 (R² = 0.8754) for irradiation of Opaque A3 at 0 mm and by y = 0.0005x + 0.9778 (R² = 0.9537) for irradiation of Opaque A3 at 8 mm.

## Discussion

The purpose of this study was to compare the depth of cure (DOC) of two types of RBCs with different opacities cured using two types of dental LED LCUs and one type of dental QTH LCUs placed close to the surface of the RBCs and at a distance from the surface of the RBCs.

First, we measured the output intensity of all 38 LCUs owned at Matsumoto Dental University Hospital with Bluephase Meter II, which has a measurable spectral range of 380 nm to 550 nm and a measurable output intensity of 300 mW/cm^2^ to 12000 mW/cm^2^. Kameyama et al. [8] reported that the output intensity of Pencure measured with an industrial spectroradiometer is 1236 ± 17 mW/cm^2^. However, this value decreased by approximately 30% to 868 ± 26 mW/cm^2^ when it was measured with the early version of Bluephase Meter. Several studies have recently shown that Bluephase Meter II produces highly reproducible results, with less variation in measured values than the early version of Bluephase Meter and fewer differences among values obtained with different Bluephase Meter II units [14-17]. Although an industrial spectroradiometer should be used to measure the output intensity of LCUs, Bluephase Meter II is considered the most reliable and reproducible of commercially available dental radiometers.

Matsumoto Dental University Hospital owns 10 Pencure 2000 LCUs. Among the LED LCUs owned by our hospital, Pencure 2000 had the highest average output intensity (1302 ± 182 mW/cm^2^) DC BlueLEX Plus had the lowest average output intensity (573 ± 158 mW/cm^2^). The output intensity of LCU often differs from the manufacturer's published values [18]. In addition, prolonged use of LCUs attenuates the output intensity [9,10,19], which is attributed to contamination of the light guide tip, in addition to other factors such as charge level, filter degradation, and reflector deterioration from long-term use [20-22]. DC BlueLEX Plus is older than Pencure 2000, and thus its initial performance is inferior to that of Pencure 2000, resulting in a lower output intensity. Although the date of purchase by the hospital is unknown, all five DC BlueLEX Plus units have been used for a long time. The light guide tips were checked for contamination before conducting the experiments, and decontamination was performed as necessary. Nevertheless, the output intensity of the LCUs was low, presumably from the internal deterioration of the instruments.

In this study, among the 10 Pencure 2000 LCUs owned by Matsumoto Dental University Hospital, we used the one with the highest output intensity (1513 mW/cm^2^) and the one with the lowest output intensity (1030 mW/cm^2^). Similarly, the unit with the highest output intensity (740 mW/cm^2^) and the unit with the lowest output intensity (320 mW/cm^2^) were selected from among the five DC BlueLEX Plus units. At an irradiation distance of 0 mm, Pencure 2000 provided a significantly higher DOC than DC BlueLEX Plus. Daugherty et al. [23] reported that the DOC of bulk-fill RBCs cured under high-intensity light is significantly greater than that of bulk-fill RBCs cured under conventional curing light. Our results reflect their findings.

QTH LCUs have a wider wavelength range than common blue LED LCUs [9]. Camphorquinone, a photoinitiator widely used for the curing of RBCs and adhesives, absorbs light in the wavelength range of 380-510 nm, which is similar to that of the irradiation light of QTH LCUs [24,25]. In contrast, blue LED LCUs generate light in a narrower wavelength range than QTH LCUs, and thus they cannot sufficiently activate some photoinitiators such as phenyl propanedione and mono acyl phosphine oxide [26]. Nevertheless, LED LCUs are preferred by many clinicians these days because of their low power consumption, wireless operation, and lightweight. In fact, many manufacturers have withdrawn their own QTH LCUs from the market [27]. However, surveys conducted in 2013 [28,29] revealed that QTH LCUs are still used in many dental clinics. In particular, QTH LCUs are sometimes built into old dental units, and thus QTH LCUs are still routinely used in many dental clinics worldwide. In fact, 5 out of the 38 LCUs in our hospital are QTH LCUs.

Four of the five QTH LCUs in our hospital are Candelux units, which are built into dental units. The unit with the highest output intensity (460 mW/cm^2^) and the unit with the lowest output intensity (320 mW/cm^2^) were used in this study. Opaque A3 has a greater opacity than Body A3. Therefore, the DOC of Opaque A3 was significantly different from that of Body A3. As shown in Figure 3, the DOC of Opaque A3 was generally smaller than that of Body A3 because the opacity of Opaque A3 was larger than that of Body A3. However, unlike the two LED LCUs, the difference in the output intensities of the two Candelux units was small, and thus attenuation, reflection, or diffusion of light inside the RBC occurred because the opacity of the RBC was high. The DOC values of the two RBCs were not significantly different because of the small difference in the output intensity of the two LCUs and the high opacity of the RBCs. 

Since the RBC used in this study (Premise) only uses camphorquinone as its photoinitiator, it is theoretically possible to absorb both the lights emitted by the LED LCU and the QTH LCU [26]. In fact, no significant difference in the depth of cure was detected between the Lowest (320 mW/cm^2^) of DC BlueLEX Plus and the Lowest (323 mW/cm^2^) of Candelux (p>0.05). Therefore, it was clarified that in RBC which uses camphorquinone as the only photoinitiator, the output intensity has a greater effect on the depth of cure than the difference in the wavelength range emitted by LCU.

In direct RBC restorations of anterior teeth, light can be applied close to the filled RBC. However, in direct RBC restorations of molars, light irradiation is generally applied from the occlusal surface in both first- and second-grade cavities. The occlusal surface of molars requires the LCU to be positioned deep in the mouth, and the presence of the cusp often prevents the approach of the LCU [30]. In addition, restorations of teeth with metal inlays or amalgam often extend to the gingival margin or just below it at the deepest depth. The average crown lengths of Japanese maxillary first bicuspids, maxillary second bicuspids, mandibular first bicuspids, mandibular second bicuspids, maxillary first molars, maxillary second molars, mandibular first molars, and mandibular second molars are 8.4 mm, 7.6 mm, 8.4 mm, 7.7 mm, 7.2 mm, 7.0 mm, 7.9 mm, and 7.2 mm, respectively. Therefore, DOC values obtained with the LCU located near and at 8 mm from the RBC were compared.

The DOC was significantly lower when the LCU was positioned at 8 mm from the RBC than when the LCU was close to the RBC, irrespective of the output intensity of the LCU. Therefore, the first null hypothesis was rejected. Ruiz-Peñarrieta et al. [18] reported that when the tip of the LCU is positioned 5 mm from the RBC, the light intensity of the LCU decreases by 26% to 44%. Malhotra et al. [22] reported that when the tip of the LCU is 5 mm from the RBC, the light intensity of LED LCUs decreases by 48.1% to 51.4%, while that of QTH LCUs decreases by 35.3% to 81.0%. Although Bluephase Meter II cannot measure the light intensity from a distance of 8 mm, the light intensity on the RBC surface is likely weakened by the same phenomenon, which may also decrease the DOC. In fact, increasing the irradiation distance to 8 mm significantly decreases the degree of conversion at the top and bottom of the RBC (thickness of 2 mm), the flexural strength of the RBC, and the micro-shear bond strength to the dentin surface, even if the output intensity of the LED LCU is high [31].

In general, dentin is high in color value and chrome but low in translucency, whereas enamel is very low in color value and chrome but high in translucency [32]. Therefore, most of the incident light penetrates through to the dentin. When direct RBC restorations are performed on molar cavities with large dentin defects, RBCs with low translucency are layered on the deep part of the cavity and RBCs with high translucency are layered on the superficial layer of the cavity [33]. If the remaining healthy dentin is colored by caries or metals in previous restoration, then it is necessary to shield the color by applying a thick layer of an opaque RBC to the deepest part of the cavity [32,34,35].

In recent years, bulk-fill RBCs have been used for deep cavities. Owing to their high transparency, bulk-fill RBCs have a greater DOC than conventional RBCs [36]. However, caries-affected dentin is difficult to remove completely [37]. Because caries-affected dentin is often darker in color than healthy dentin, its color cannot be masked by bulk-fill RBCs alone [33]. Therefore, RBCs with low translucency should be used on a case-by-case basis to achieve esthetic restorations. In this study, the DOC of Opaque A3 was smaller than that of Body A3, regardless of the type of LCU and irradiation distance. The LCU with the higher output intensity provided a greater DOC than the LCU with the lower output intensity. Therefore, the second null hypothesis was also rejected. These results indicate that LCUs with a high output intensity should be used to cure RBCs with low translucency.

In general, radiant energy of 16 to 24 J/cm^2^ is required to polymerize RBCs with a thickness of 2 mm [38]. This energy (E) is calculated by multiplying the irradiance (I) by the LCU (mW/cm^2^) by the irradiation duration (T) [30]. Therefore, theoretically, a DOC of 2 mm can be achieved by irradiating light for a very long time even at a low output intensity, although irradiating deep cavities for a long time may damage pulp tissue because of the large temperature increase [39, 40]. In this study, the DOC of Opaque A3 was less than 2 mm even after 30 s of light irradiation with Pencure 2000. Therefore, when applying RBCs with strong opercular characteristics to the cavity floor, it is recommended to layer the RBC as thinly as possible and irradiate it with light of sufficient intensity for a long period of time.

Unfortunately, at the time of this study, the LCUs were not equipped with a special wrapping cover. However, most departments now install a new wrapping cover before using the LCU. Although the cover may slightly decrease the output intensity [41], it not only prevents the RBC and adhesive on the tip of the light guide from weakening the output intensity but also serves as an important standard precaution [42]. Before using the LCU, it may be necessary to use a dental radiometer to check whether sufficient light intensity is supplied to the RBC.

## Conclusions

The conclusions of this study are as follows: (1) The DOC of the RBCs was larger when the output intensity at the tip of the light guide was higher for the same model of LCU; (2) The DOC was smaller when the LCU was positioned 8 mm from the RBC surface than when the LCU was close to the RBC surface; (3) The DOC of the opaque-shade RBC was smaller than that of the body-shade RBC when the same LCU was used.
